# Another Resolution

**Published:** 1882-04

**Authors:** 


### ANOTHER RESOLUTION.

At the meeting of the Michigan Dental Association in March, 1881, Dr. A. T. Metcalf, of Kalamazoo, offered some resolutions on the subject of prosthetic dentistry that were pretty sharply criticised by a large number in the profession. The resolutions, on the face, at least, conveyed the idea that the making and inserting of artificial teeth was a matter of little importance, and really beneath the attention and serious consideration of the dental surgeon. That the resolutions referred to did not fairly represent the views of Dr. Metcalf may be rightly inferred from the following, which he offered at the annual meeting of the same body held a few days ago in the city of Detroit. The following is from the proceedings of that meeting:

"Dr. Clawson occupying the chair, Dr. Metcalf moved to take from the table a resolution, offered by him at the last session, providing for the abolition of the chair of mechanical dentistry at the University. Carried, and the following substitute was offered:

*Whereas,* That part of our practice known as mechanical dentistry, when so conducted as to give to patients the highest attainable results, has become so intricate and complicated that it can not be properly learned in the time now devoted to the teaching of it in our Dental College; and

*Whereas,* In consequence of the insufficient instructions now given in it tends to cheapen, belittle and degrade it; therefore,

*Resolved*, That the officers of the Association and the visitors to the Dental Department of the University of Michigan are hereby instructed to make all proper efforts to have mechanical dentistry taught as a distinct calling, and when students have become proficient in its various departments that they be entitled to receive a certificate of qualification to practice it, regardless of their qualification to practice dental surgery.

The resolution was laid on the table, and the Association then adjourned."

This presentation of the subject, while it may be in some slight degree premature, cannot in its intent, and even in its execution, be otherwise received than favorably by those who wish to see the highest attainments made in the profession--and especially in the prosthetic department, which has been for these many years at so low an ebb. May the subject receive earnest attention and full consideration, till the much needed improvement is made.

